# Education for Ward Nurses Influences the Quality of Inpatient's Bowel Preparation for Colonoscopy

**DOI:** 10.1097/MD.0000000000001423

**Published:** 2015-08-28

**Authors:** Yoo Jin Lee, Eun Soo Kim, Kyung Sik Park, Kwang Bum Cho, Byoung Kuk Jang, Woo Jin Chung, Jae Seok Hwang

**Affiliations:** From the Division of Gastroenterology and Hepatology, Department of Internal Medicine, Keimyung University School of Medicine, Daegu, Korea.

## Abstract

Although adequate bowel preparation is a prerequisite for colonoscopy, preparation among inpatients is often suboptimal. This study aimed to evaluate the impact of ward nurse education on the quality of bowel preparation of inpatients.

A prospective, double-blinded, non-randomized, controlled study was performed. Expert endoscopists provided enhanced education to nurses who belonged to an “educated ward” followed by training that was repeated every week for 1 month. The primary outcome was the quality of the bowel preparation, which was based on the Ottawa Bowel Preparation Scale (OBPS). Patient compliance and their subjective feelings and the factors affecting inadequate bowel preparation were also analyzed.

One hundred three inpatients in the educated ward and 102 patients in the control ward were enrolled. Baseline data were comparable between the 2 wards. The mean values of the total OBPS scores were 4.42 ± 2.23 and 6.15 ± 2.38 in the educated and control wards, respectively (*P* < 0.001). The rate of poor preparation (OBPS ≥ 6) in the educated ward was significantly lower than that in the control ward (31.1% vs 58.8%, *P* < 0.001). Compliance with preparation and diet instructions in the educated ward was superior to that in the control ward (*P*<0.001). Control patients were more likely to be anxious before colonoscopy (*P* < 0.001), whereas patients in the educated ward showed higher level of satisfaction (*P* = 0.001) and better sleep quality (*P* < 0.001). A lack of ward nurse education (OR 2.365, *P* = 0.025), constipation (OR 6.517, *P* < 0.001), and insufficient water ingestion (OR 2.044, *P* = 0.042) were independently associated with inadequate bowel preparation among inpatients.

Ward nurse education effectively improved the quality of bowel preparation, and relevant colonoscopic outcomes among inpatients. Additional efforts are needed to control constipation and to encourage additional water ingestion in inpatients for better bowel preparation.

## Abstract 2

Supplemental Digital Content is available in the text

## INTRODUCTION

As the diagnostic accuracy of colonoscopy largely depends on the quality of bowel preparation, effective bowel preparation is essential. The quality of bowel preparation among inpatients is poorer than that among outpatients.^1,2^ One prior study revealed that the rate of adequate bowel preparation was only 50% in hospitalized patients.^3^ Consequently, the economic burden of inadequate bowel preparation for inpatients is considerable because of failed colonoscopy and incomplete examination, ultimately leading to repeated colonoscopies; this, in turn, depletes the already limited endoscopic resources and increases health care expenditures.^1^

During bowel preparation, patients are required to cooperate actively with respect to the prescribed purgative and diet instructions. Many patients, however, often misunderstand these complicated requirements, and as a result, it is a great challenge for them to complete their bowel preparation successfully.^4^ Hence, several studies have suggested educational tools to enhance a patient's comprehension and compliance for better bowel preparation quality.^5–7^ However, most studies in the area of education for bowel preparation have focused on outpatients and not on inpatients.

The additional education for nurses has been attempted in diverse hospital settings to provide optimal care to inpatients, and most of these efforts resulted in improved clinical outcomes.^8,9^ Educated nurses reported the resolution of their “uncertainty and lack of confidence” after the education was provided, which allowed them to provide effective care and appropriate information to their patients.^9^

Therefore, we aimed to investigate the effect of nurse education on the quality of bowel preparation for inpatient colonoscopy. We also evaluated colonoscopy outcomes as well as the compliance and subjective feelings of the patients.

## MATERIALS AND METHODS

This study was a prospective, double-blinded, nonrandomized controlled trial conducted at a tertiary hospital in South Korea. The participants were assigned to the educated ward or to the control ward. The study protocol was approved by the institutional review board of our institution. Written informed consent was obtained from all participants. This study was published in the www.ClinicalTrials.gov registry (NCT01911026).

### Participants and Assignments

Between July 2013 and January 2014, patients aged 19 to 80 years who were admitted to 2 gastroenterology wards of the hospital and who were scheduled for a nonurgent colonoscopy were recruited. Patients were excluded for the following reasons: known hypersensitivity to polyethylene glycol (PEG); severe congestive heart failure (New York Heart Association [NYHA] grade III or grade IV); severe renal insufficiency (creatinine clearance <30 mL/minutes); hemodynamic instability; suspected intestinal obstruction or perforation; compromised swallowing reflex or altered mental status; and, among women, pregnancy or lactation. Patients undergoing an unscheduled colonoscopy or who declined to participate were also excluded.

After they agreed to participate, the patients were consecutively admitted to one of the wards as rooms became available. Any systematic allocations by the investigator were not allowed during the assignments of the wards. Patients in the educated ward received explanations of the steps required for bowel preparation from intensively educated nurses, whereas patients in the control ward received the standard explanations from nurses who had received no enhanced education.

At the beginning of this study, we performed a preliminary test with pilot study samples (each group consisted of 30 patients) to clarify any differences between the patients in the 2 wards. As a result, no significant differences were observed in terms of the patient demographics, the quality of bowel preparation, colonoscopy outcomes, subjective feelings, or compliance with the instructions (Supplementary Table 1, http://links.lww.com/MD/A389).

Nurses in both wards were instructed not to reveal their group assignments to any of the participants or to the investigators. Thus, all participants, interviewers, physicians, and colonoscopists were blinded to the group allocations during the study period.

### Bowel Preparation and Diet Protocol

The methods of bowel preparation and diet restrictions were equally applied to all patients. The patients were prescribed low-residue diets at least 2 days before the scheduled colonoscopy; they were also advised not to ingest high-fiber foods. On the day before the colonoscopy, the patients were provided a soft diet for dinner before 6 pm, and after that time, only clear water was allowed. Then, 2 l of PEG + ascorbic acid (Asc) (Coolprep, Taejoon Pharm. Inc., Seoul, Korea), which contained 100.0 g PEG, 7.5 g sodium sulfate, 2.7 g sodium chloride, 1.0 g potassium chloride, 4.7 g Asc, and 5.9 g sodium ascorbate per l, was ingested at the rate of 250 mL every 10 minutes. For colonoscopies performed in the morning, a split-dose bowel preparation regimen was followed: ingestion of a half-dose of purgative at 8:00 pm on the day before the procedure and the remaining 1 l on the morning of the procedure. For colonoscopies performed in the afternoon, patients were asked to consume a full dose (2 l) of PEG + Asc between 6:00 and 8:00 am on the day of procedure. Additionally, patients were instructed to drink clear water until their stools did not show the presence of any brown effluent. All colonoscopies were performed between 2 and 8 hours after the purgative intake was complete.

### Intervention: Nurse Education

Two expert colonoscopists (ESK and YJL) provided enhanced education, which consisted of a leaflet and a lecture, to nurses who belonged to the educated ward. The enhanced education explained the importance of bowel preparation and the possible adverse effects during bowel preparation (eg, nausea, vomiting, abdominal pain, headache, electrolyte imbalance, and others) for the purpose of reducing a patient's anxiety. This training also explained the precolonoscopy diet and the rationale for this diet, the instructions for completion of the purgative, and the importance of drinking additional water as needed. During the lecture, nurses were allowed to pose any questions. The leaflet was placed on the wall of the educated ward, and brief review training sessions for nurses were repeated every week for 1 month. No additional education was provided to nurses who were assigned to the control ward.

### Outcomes

The primary outcome was the quality of bowel preparation as measured by 5 expert colonoscopists (KSP, KBC, BKJ, WJC, and JSH) who each had experience with over 2000 colonoscopies. Factors affecting inadequate bowel preparation, as assessed by the proportions of inadequate bowel preparation were also identified. The secondary outcomes were the subjective feelings of the patients during the preparation, their compliance with the instructions and colonoscopic outcomes such as the adenoma detection rate (ADR) and the rate of incomplete colonoscopy.

### Assessments of the Quality of Bowel Preparation

The Ottawa Bowel Preparation Scale (OBPS) was used to evaluate the quality of bowel preparation. An assessment was performed on the overall fluid volume (0 = small, 1 = moderate, and 2 = large) of the entire colon as well as its cleanliness, which was rated from 0 to 4 (0 = excellent, 1 = good, 2 = fair, 3 = poor, and 4 = inadequate), for the right colon, mid-colon, and rectosigmoid colon. The total OBPS score was calculated as the sum of the overall fluid volume and each of the segmental scores and ranged from 0 to 14; a higher score indicates poorer preparation.^10^ During the study period, the OBPS usage manual, which contains representative colonoscopic images of each score, was posted to the wall of the endoscopic procedure room. Prior to patient enrollment, the participating colonoscopists received standard instructions on how to use the bowel preparation scale to reduce possible interobserver variation. Then, a pilot study was performed to ensure interobserver agreement for the rating of the preparation quality using 20 colonoscopies. As a result, a high level of interobserver agreement was achieved (k = 0.87).

All colonoscopies were performed with conventional video endoscopes between 10:00 am and 4:00 pm. To ensure that the colonoscopists were blinded, information regarding the patient's ward was not revealed to the colonoscopists; to this end, we removed the room number and the ward of the patient from the colonoscopy report form and from the patient's personal items. Colonoscopists were instructed not to ask the patients about the wards to which they were admitted. Only 1 endoscopy nurse who was not involved in the study identified a patient's ward information. Patients had the choice of conscious sedation or not during the procedure. However, the decision was ultimately determined by the individual colonoscopist after consideration of the patient's clinical status. Immediately after the colonoscopy, the colonoscopists recorded the OBPS score which was applied when scope withdrawal and the detailed outcomes such as cecal intubation time, withdrawal time, and colonoscopic findings. ADR was defined as the proportion of colonoscopies with at least 1 adenoma was detected.^11^

### Assessment of the Patients’ Compliance and Subjective Feelings

On the day of the colonoscopy, 1 trained nurse who was unaware of the allocated groups of the patients interviewed all patients before their procedure. Then, the collected data were immediately entered into a prepared form in a database. The information obtained through interview was as follows: patient's characteristics, the amount of remaining purgative, how closely the patient followed the dietary restrictions, how strictly the patient followed the instructions for the purgative, whether the patient ingested additional water if needed, the degree of sleep disturbance, patient's anxiety and satisfaction with preparation, adverse events during bowel preparation, willingness to repeat the same preparation, and completion time of purgative ingestion.

Constipation was defined as having at least 2 of 6 specific bowel symptoms including straining, lumpy or hard stools, incomplete evacuation, sensation of anorectal obstruction, manual maneuvers for defecation, and ≦3 defecations/week.^12^ Compliance with the dietary and preparation instructions was classified as high (over 50%) or low (below 50%). The question regarding “additional water ingestion if needed” was assessed with “yes” or “no” answer. Similarly, the presence of sleep disturbance and willingness to repeat the same preparation were also indicated with a “yes” or “no” response.

According to previous reports, patient's anxiety and satisfaction were rated on a 5-point scale (very low, low, moderate, high, and very high).^7^ The preparation-to-colonoscopy (PC) interval, which was defined as the time from the completion of purgative intake to the start of the procedure, was measured in all patients.^13^

### Statistical Analysis

Calculation of the sample size was based on the result of an earlier study that determined an adequate bowel preparation of inpatients as 50%.^3^ To obtain a 20% improvement (from 50% to 70%) in the occurrence of adequate bowel preparation in the nurse educated ward, a sufficient sample size per group was found to be 90 at a 2-tailed 5% significance level with a power of 80%. Considering a 10% dropout rate, we estimated that a minimum of 100 patients per group were needed.

The categorical variables were analyzed by Chi-square tests or Fisher exact tests. Student's *t*-tests were used for continuous variables. A logistic regression analysis was performed to assess the factors associated with inadequate bowel preparation (OBPS ≥ 6). Variables that reached a *P* value of <0.05 by univariate analysis were analyzed in a multiple logistic regression model. A *P* value of <0.05 was considered statistically significant. Statistical analysis was performed using SPSS software (Ver. 20.0; SPSS Inc., Chicago, IL).

## RESULTS

**FIGURE 1:**
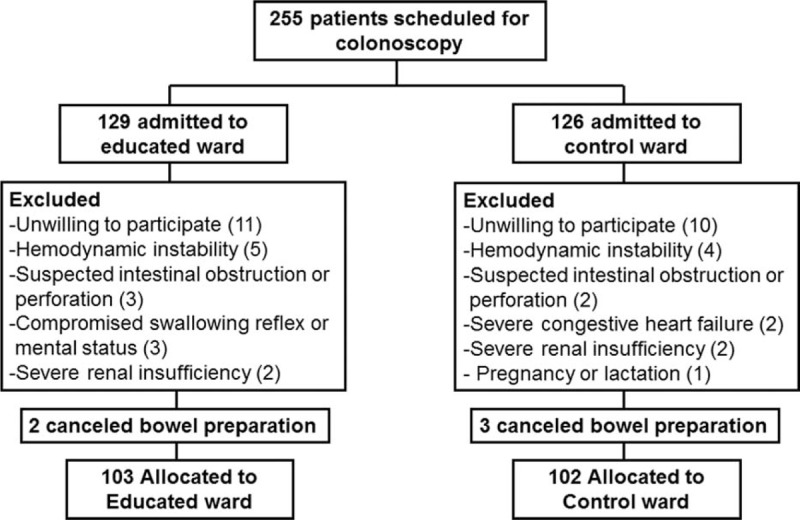
Enrollment flow chart of the patients.

A total of 255 patients who were scheduled for a colonoscopy in 2 gastroenterology departments during the study period were screened. Of the 129 who were admitted to the educated ward and of the remaining 126 who were admitted to the control ward, 45 who met the exclusion criteria were excluded. Five patients canceled their colonoscopy appointment. Finally, 103 inpatients in the educated ward and 102 patients in the control ward underwent colonoscopy. Figure 1 depicts the flow chart of the patient enrollment. These 2 groups showed similar baseline characteristics at inclusion (Table 1).

**TABLE 1:**
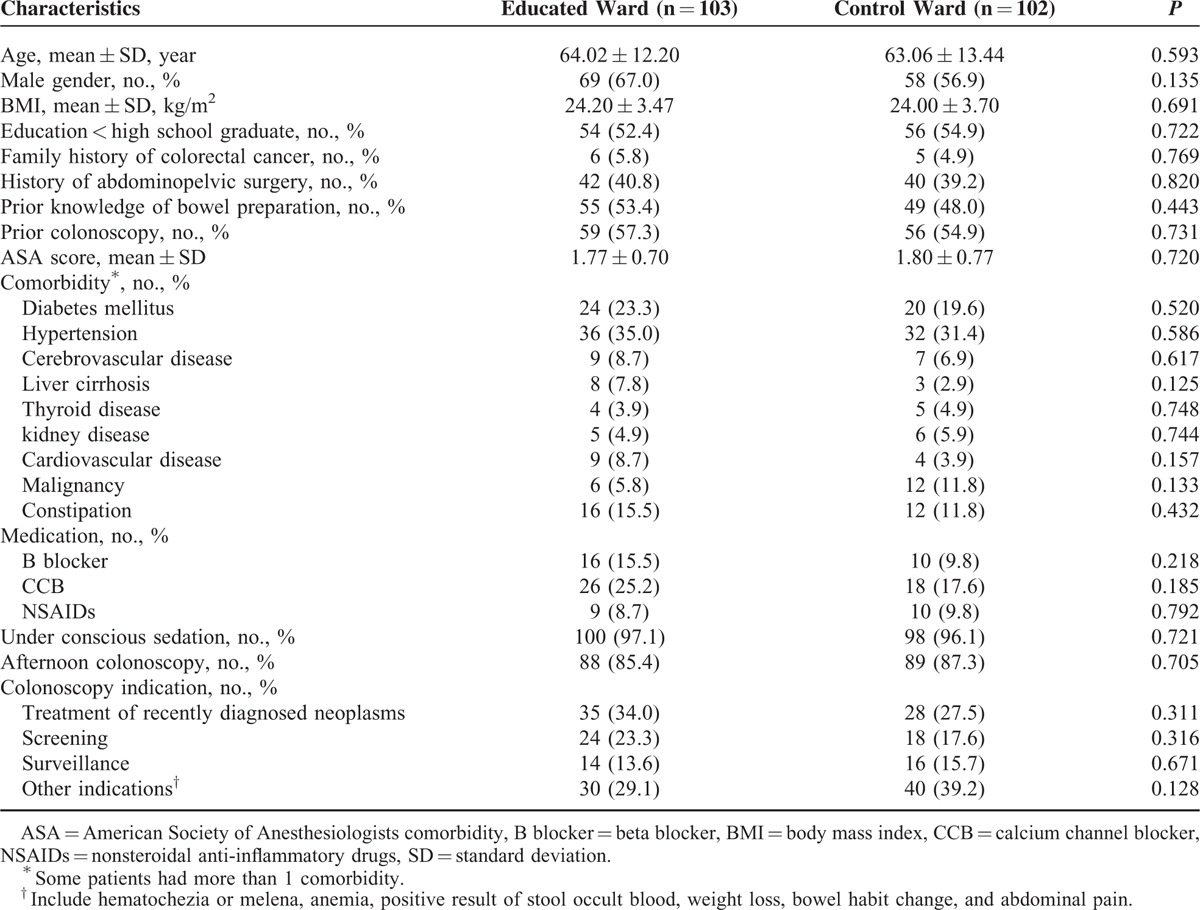
Baseline Characteristics of Patients (n = 205)

### Clinical Outcomes and Efficacy of Bowel Preparation

The colonoscopy outcomes of each group are presented in Table 2. The mean cecal intubation time in patients in the educated ward was shorter than that of patients in the control ward (6.21 ± 3.72 vs 8.06 ± 6.68 minutes, *P* = 0.016), whereas the withdrawal times were not different between the 2 groups (*P* = 0.610). The mean PC interval was also shorter in the educated ward than in the control (4.46 ± 1.72 vs 5.35 ± 2.21 hours, *P* = 0.001). Notably, the ADR was significantly higher in the educated ward compared with the control (58.3% vs 43.1%, *P* = 0.030) although it lost its significance after eliminating patients who were referred to the hospital for the treatment of recently diagnosed colonic neoplasms. No differences were observed between the 2 groups in terms of the cecal intubation rates, causes of incomplete colonoscopy or average doses of sedative agents. None of the patients reported severe adverse events.

**TABLE 2:**
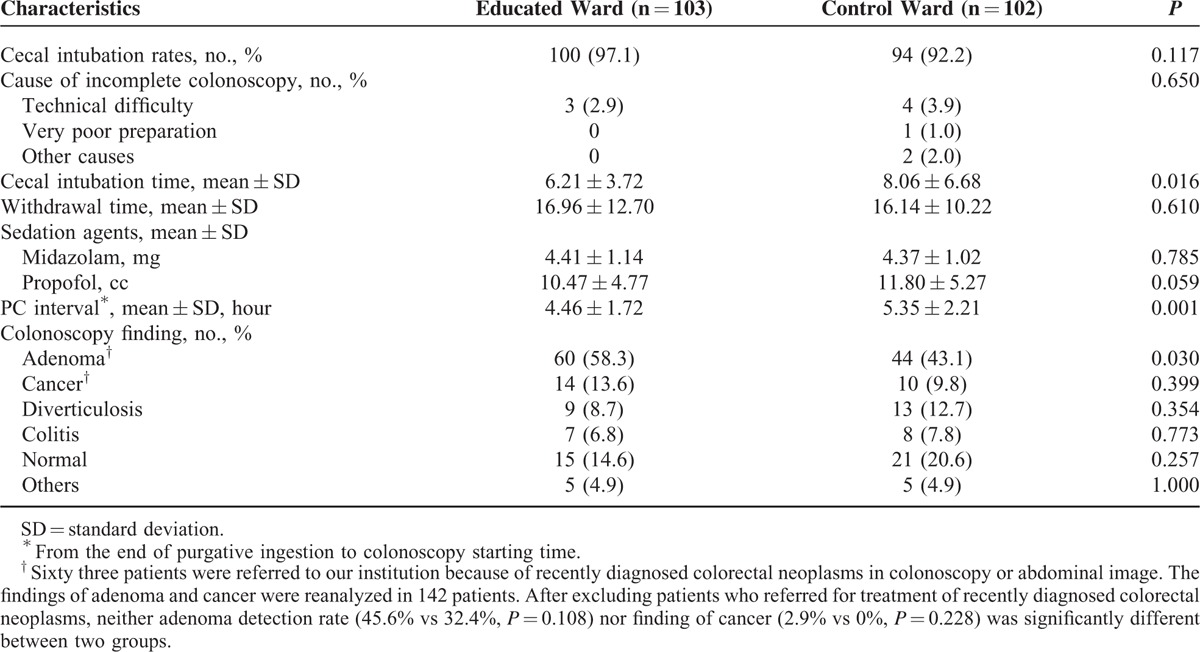
Comparison of Colonoscopy Outcomes

Table 3 shows the distributions of the OBPS scores. The overall fluid volume and cleanliness scores for each segment were superior in the educated ward. More patients in the educated ward were found to have a lower total OBPS score, which suggests a better quality bowel preparation in the educated ward (4.42 ± 2.23 vs 6.15 ± 2.38, *P* < 0.001). The proportion of patients with inadequate bowel preparation (OBPS ≥ 6) was significantly lower in the educated ward compared with the control (31.1% vs 58.8%, *P* = 0.001).

**TABLE 3:**
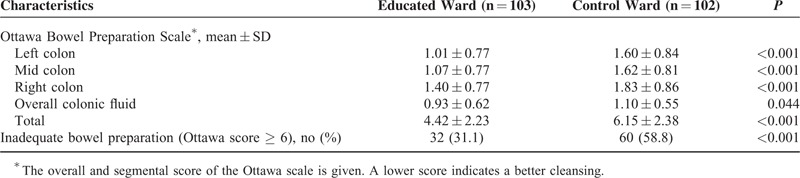
Comparison of Bowel Preparation Quality

### Subjective Feelings and Compliance

Only 20.4% of patients in the educated ward responded that their sleep quality was worse than usual, whereas 44.1% of patients in the control ward did (*P* < 0.001). The reported anxiety was lower in patients in the educated ward than in control (*P* < 0.001). Patients in the educated ward also reported better satisfaction than patients in control (*P* = 0.001). However, no differences were observed between the 2 groups in the reported preparation-related adverse symptoms and in the proportion of patients who were willing to undergo the same preparation again (Table 4).

**TABLE 4:**
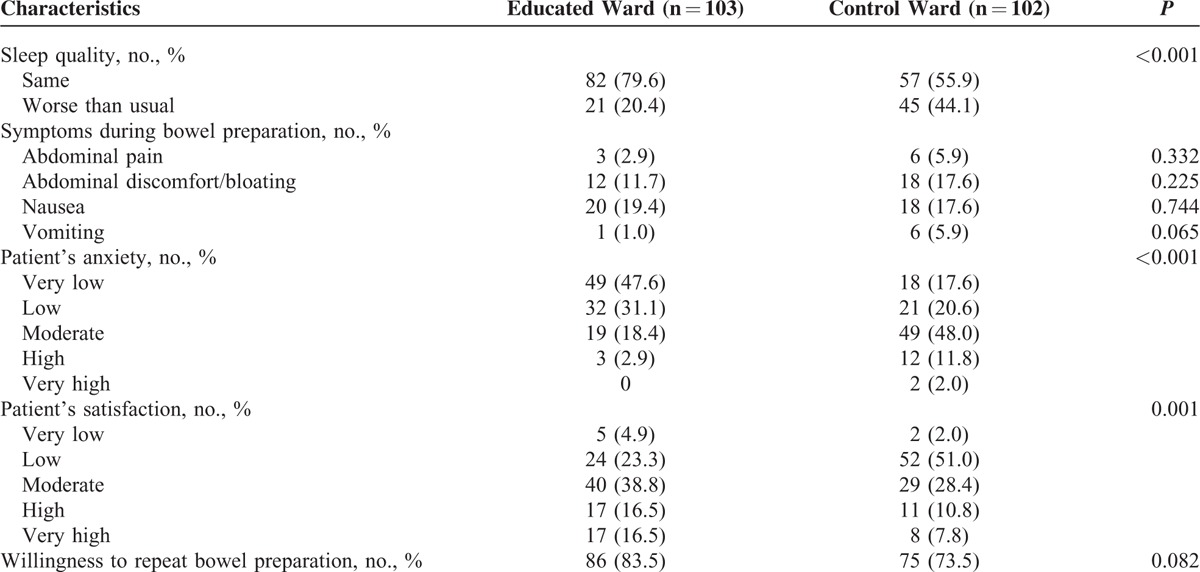
Comparison of Patient's Subjective Feelings

Table 5 presents the comparisons of the compliance of the patients in both groups. All variables concerning the compliance of the patients during the bowel preparation were significantly better in the educated ward than in control; the proportion of patients who ingested more than 80% of the purgative was higher in the educated ward than in control (98.1% vs 89.2%, *P* = 0.009). Preparation instructions were also more closely followed in the educated ward than in control (92.2% vs 60.8%, *P* < 0.001). Similarly, the proportion of patients who complied with the dietary instructions was higher in the educated ward than in control (83.5% vs 35.3%, *P* < 0.001). With regard to the recommendations of additional water intake as needed, more patients in the educated ward followed this recommendation than did patients in control (63.0% vs 26.0%, *P* < 0.001).

**TABLE 5:**
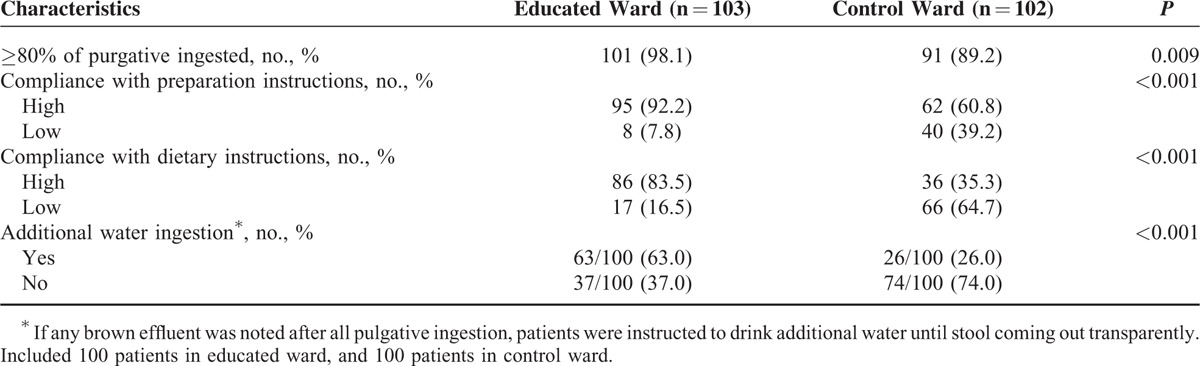
Comparison of Patient's Compliance During Bowel Preparation

### Factors Affecting the Quality of Bowel Preparation

Of the 205 patients who were enrolled in this study, 92 (44.9%) showed inadequate bowel preparation (OBPS ≥ 6). In univariate analysis, the presence of constipation (*P* < 0.001), a lack of ward nurse education (*P* < 0.001), low compliance with preparation instructions (*P* = 0.002) and dietary instructions (*P* = 0.001), and no additional water ingestion (*P* < 0.001) were revealed as factors related to inadequate bowel preparation (Table 6). A multivariate analysis showed that the followings were independent factors associated with inadequate bowel preparation: the presence of constipation (odds ratio [OR] 6.517, 95% confidence interval [CI], 2.299–18.473, *P* < 0.001), a lack of ward nurse education (OR 2.365, 95% CI, 1.114–5.018, *P* = 0.025), and no additional water ingestion (OR 2.044, 95% CI, 1.026–4.070, *P* = 0.042) (Table 7).

**TABLE 6:**
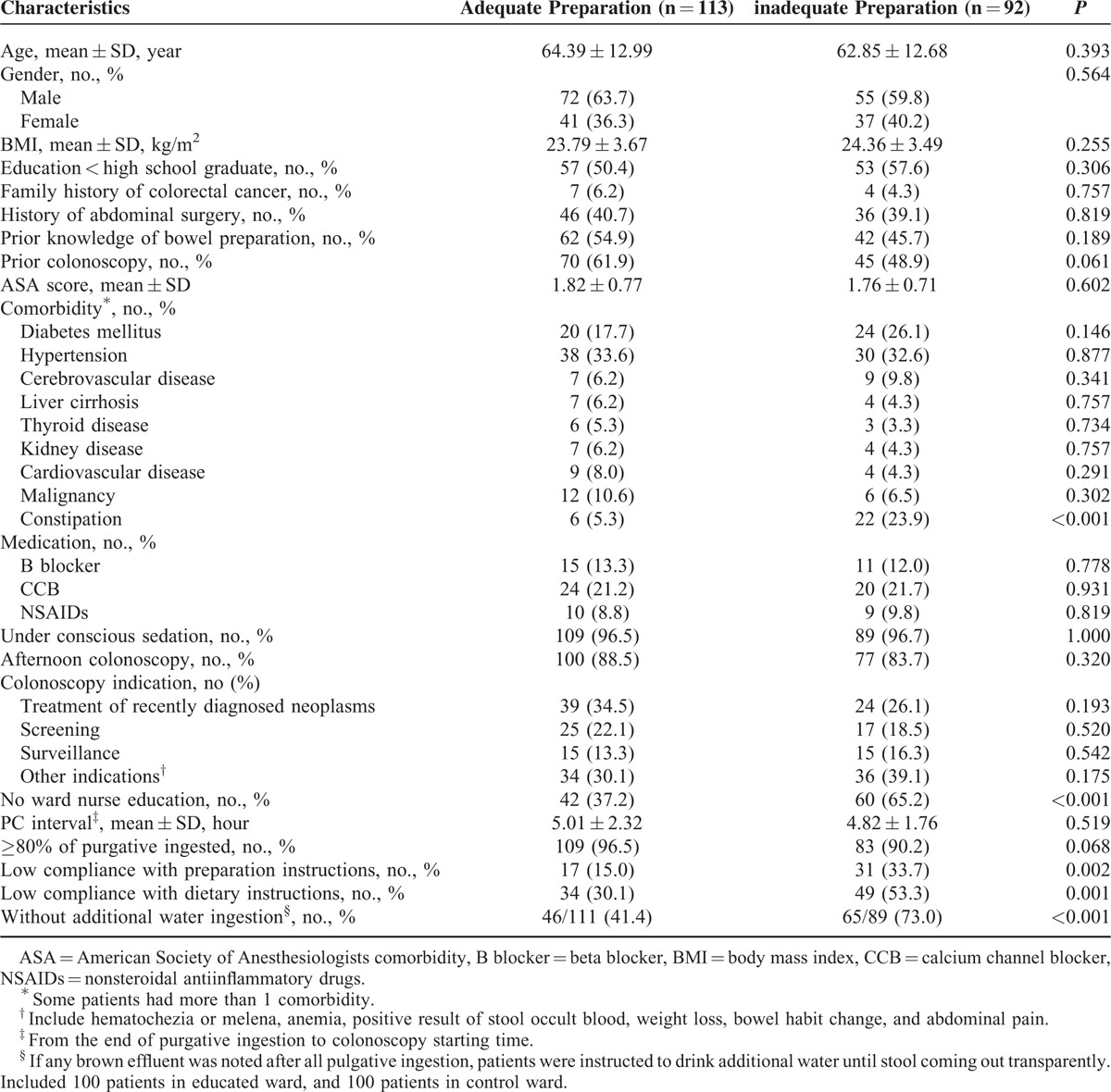
Univariate Analysis of Factors Associated With Inadequate Bowel Preparation (Ottawa Score ≥6)

**TABLE 7:**
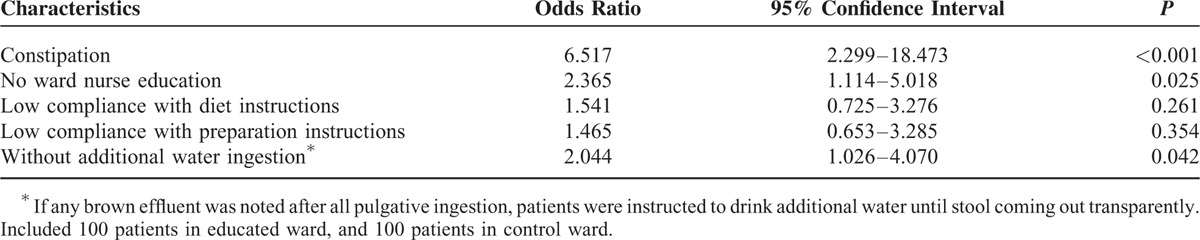
Multivariate Analysis of Factors Associated With Inadequate Bowel Preparation (Ottawa Score ≥6)

## DISCUSSION

Inpatient status is an independent predictor of inadequate bowel preparation.^3,14,15^ Several hypotheses have been proposed as to the causes of inadequate preparation in this population. Hospitalization due to acute illness or aggravation of chronic illness often results in poor adherence to preparation instructions.^3^ The characteristics of inpatients, including old age, a decreased level of physical activity and certain comorbidities, are closely associated with a diminished or altered bowel motility that inhibits satisfactory preparation.^1^ Additionally, shared facilities limit the inpatient's access to the toilet, which may result in failed preparation in some patients.^2^ Thus, additional intensive strategies have been required for satisfactory bowel preparation in inpatients.

Given that a ward nurse provides frontline primary care for each hospitalized patient in terms of diet, medications, and periprocedural management, the role of the bedside nurse may be crucial for the bowel preparation of inpatients. However, no studies have evaluated the role of ward nurses in colonoscopic bowel preparation among inpatients.

We hypothesized that education for ward nurses can be a useful strategy to improving the quality of bowel preparation among inpatients as well as their understanding of its importance. The results of this study support the substantial influence of the ward nurse on bowel preparation among inpatients. Patients who received instructions from educated ward nurses demonstrated greater compliance with instructions, higher satisfaction, and better quality of bowel preparation. Anxiety was also reduced in patients in the educated ward. The most likely explanation for this beneficial effect of nurse education on bowel preparation is that patient satisfaction with hospital care largely depends on patient satisfaction with nursing care^16^ and that patient perception of nursing care has been associated with positive-patient outcomes.^17^ Abbott^18^ reported that noncompliant behavior might be attributed to confusion, disappointment, misunderstanding, and fear and that compliance increases when patients understand the rationale for the procedure, the steps required for the procedure, and the potential adverse effects. We believe that frontline nurses who better understand the importance of the quality of bowel preparation for colonoscopy, the rationale of the instructions for diet and purgatives, and the possible complications of the purgatives can provide more effective care and can deliver relevant information to individual patients. Better educated nurses can also foster the compliance of patients with regard to the procedure, leading to better bowel preparation in clinical practice.

One previous study suggested that educational interventions for medical staff had no impact on the preparation quality and colonoscopy success rates in inpatients.^1^ This failure in the previous study might be attributed to insufficient educational effect; they provided only single educational session. In contrast, our enhanced education consisted of initial lectures with a question and answer session followed by periodic brief review sessions. Nurses in the educated group could also review the key messages of the educational at any time by consulting the leaflet displayed on the wall of the ward.

Several studies support the benefit of preprocedural information on relieving anxiety among patients.^19–21^ Because colonoscopy is an anxiety-provoking procedure that has been linked with pain and complications, it is expected that informed patients would exhibit reduced anxiety and improved tolerability to the procedure.^20^ In this study, the patients’ self-reported anxiety was significantly lower in the educated ward compared with the control, indicating the effectiveness of nurse education in providing the patients with information on the procedure.

Several predictors of inadequate preparation have been suggested, such as older age, chronic constipation, the use of concurrent medications, prior abdominal surgery, and comorbidities.^15,22^ However, only a few studies have identified such predictors that are specific to inpatients.^23^ Therefore, we evaluated factors associated with inadequate bowel preparation among inpatients. In multivariate analysis, education of ward nurses reduced inadequate bowel preparation, which confirms the efficacy of this strategy for the improvement of bowel preparation quality among inpatients.

Constipation is a well-known predictor of unsatisfactory preparation,^6,15^ as shown in our results with an OR of 6.517 (Table 7). Delayed colonic transit time due to constipation that is resistant to purgatives may result in inadequate bowel cleansing.^24^ We also assumed that several environmental changes may aggravate preexisting constipation when patients were hospitalized, such as decreased ambulation and limited access to the toilet. Our study also found that the ingestion of additional water until the stools were clear was inversely correlated with inadequate bowel preparation. Based on the results showing that patients with brown rectal effluent on presentation had approximately a 50% higher risk of suboptimal preparation, it would be advisable to verify the color of a patient's effluent and recommend the ingestion of additional water if necessary.^4^

There have been several validated bowel preparation scales including the Aronchick scale, the OBPS, and the Boston bowel preparation scale (BBPS). The Aronchick scale is the first validated scale to make global assessment of the entire colon, without provision for assessing individual segments.^25^ Unlike Aronchick scale, both OBPS and BBPS use segmental assessment of bowel cleansing then summation of the segmental scores. The OBPS is designed to rate 3 region of the colon independently and overall quantity of fluid in the entire colon before suctioning.^10^ The BBPS consists of aggregate of 3 colonic segment scores which are rated during colonoscopy withdrawing, after washing and suctioning of fluids.^26^ Thus, the effort to remove residual fecal materials and liquids are important in OPBS, whereas it is not in the BBPS.^27^ Both OBPS and BBPS has a shortcoming of possibility of false acceptable global score, for instance, high scores in 1 segment of colon may mask an inadequate bowel preparation in another part of the colon.^28^ Although the choice of a bowel preparation scale has a strong influence on the outcome of a bowel preparation study, there is no ideal validated scale yet.^27,28^ Further researches are necessary to identify more reliable and feasible bowel preparation scale in clinical practice.

Our study has certain strengths. First, not only were various clinical factors that may negatively impact the quality of bowel preparation studied, but other important aspects, including a patient's subjective feelings and compliance during the preparation, were also assessed in this study. Second, we used the OBPS, which is a validated and widely adapted scale for evaluating the quality of bowel preparation. Moreover, we tried to reduce the interobserver variability based on a pilot test comparing OBPS ratings among the colonoscopists. Third, to avoid the possibility of selection bias, we unified the preparation regimen including the prescribed purgative agent, the timing, dose, and method for purgative ingestion, and we controlled the PC time within the range of 2 to 8 hours. Lastly, we identified factors that are related to inadequate bowel preparation in inpatients. These factors may be helpful in determining inpatients who might be at greater risk for inadequate bowel preparation.

The current study also has several limitations. First, this was not a randomized study. The allocation of patients to each ward could not be randomly controlled due to administrative constraint at the hospital. Therefore, some unexpected confounding factors resulting from selection bias may affect the process and results of this study. However, to overcome the shortcomings associated with nonrandomization, we strictly controlled the blinding of the patients and investigators during the prospectively designed study periods. Through a pilot study, we also verified that the prestudy patient characteristics and study outcomes were well balanced between both groups. Second, this study was conducted in a single institution, and we only included patients who were hospitalized in the gastroenterology department. Therefore, care should be taken in the generalization of these findings. Third, we did not evaluate the duration of the effect of education.

Our findings suggest the importance of enhanced education for frontline nurses that can be easily applied in a real clinical practice with lower demand on resources. Thus, clinicians should consider the application of ward nurse education in each healthcare facility for achieving better bowel preparation in inpatient's colonoscopy.

In conclusion, enhanced education of ward nurses is effective because it leads to a better quality of bowel preparation and improves the patients’ subjective feelings and compliance in inpatient colonoscopy. Additional efforts to control constipation and to encourage additional water ingestion are needed to improve quality of bowel preparation among inpatients.
